# Brief mindfulness intervention prior to simulated central venous access device insertion: A pilot randomised feasibility study

**DOI:** 10.1016/j.ccrj.2026.100198

**Published:** 2026-07-09

**Authors:** Denis O'Dwyer, Ashwin Subramaniam, Dean McKenzie, Tony King, Laven Padayachee

**Affiliations:** a Department of Intensive Care, Epworth Hospital, Epworth Healthcare, Richmond, VIC, Australia; b Department of Intensive Care, Dandenong Hospital, Monash Health, Dandenong, Victoria, Australia; c Australia and New Zealand Intensive Care Research Centre, School of Public Health and Preventive Medicine, Monash University, Melbourne, VIC, Australia; d Office for Research, Epworth HealthCare, Melbourne, Australia

**Keywords:** Mindfulness, Procedural performance, Randomised pilot study, Physiological arousal, Cognitive workload

## Abstract

**Background:**

Acute stress impairs attention and procedural performance in high-acuity environments. Whether brief mindfulness-based interventions are feasible and affect performance in intensive care unit (ICU) simulation training is unknown.

**Objectives:**

To evaluate the feasibility of a brief mindfulness-based intervention before simulated ultrasound-guided central venous access device (CVAD) insertion.

**Design, setting and participants:**

Single-centre pilot randomised feasibility study at a tertiary ICU simulation centre. Twelve of thirteen ICU registrars with prior CVAD experience were randomised 1:1 to mindfulness or an active control.

**Intervention:**

A 3-min recorded mindfulness meditation focused on breath awareness and attentional regulation, or a time-matched active control (standardised observation chart review), delivered immediately prior to simulation.

**Main outcome measures:**

Feasibility thresholds: recruitment ≥80%, adherence ≥90%, data completeness ≥95%, and assessor blinding. Exploratory outcomes included procedural performance (checklist, global rating scale, and total score), physiological arousal (heart rate and blood pressure), psychological state anxiety (STAI-State), confidence, and cognitive workload (NASA-TLX).

**Results:**

All feasibility thresholds were met (recruitment [92%], adherence, and data completeness [100%, respectively]; blinding maintained). Mindfulness produced greater transient reductions in heart rate (−12.4 vs −5.3 beats/min) and systolic blood pressure (−13.5 vs −3.7 mmHg). STAI-State decreased in the mindfulness group but was unchanged in controls. Postsimulation confidence was higher in the mindfulness group (mean 13.7 vs 11.8). Procedural performance was numerically lower in the mindfulness group across all domains (total score 41.3 vs 52.0), and critical procedural errors were more frequent (4/6 vs 1/6). Confidence intervals were wide throughout, reflecting the small sample.

**Conclusions:**

A brief preprocedural mindfulness intervention was feasible and transiently reduced physiological arousal and state anxiety, but did not improve procedural performance; scores were numerically lower in the mindfulness group and critical errors were more frequent, though chance imbalance cannot be excluded given the small sample. These findings support progression to a larger, multicentre, adequately powered trial.

## 1 Introduction

Procedures in critically ill patients are inherently stressful. They are performed in environments characterised by time pressure, uncertainty, physiological instability, and high cognitive load, where errors may have immediate consequences for patient safety. Acute stress impairs attention, working memory, and decision-making, with downstream effects on procedural performance.^1^^,^^2^ However, the relationship between stress and performance is not linear. The Yerkes–Dodson law describes an inverted-U relationship wherein both insufficient and excessive arousal impair performance, with an optimal zone that varies by task complexity and individual characteristics.^3^ In critical care, this is particularly relevant: proceduralists must maintain sufficient arousal to sustain vigilance and fine motor coordination, while avoiding the attentional narrowing and working memory degradation that accompany excessive stress.

Central venous access device (CVAD) insertion is one of the core procedures in intensive care unit (ICU) practice. Safe and effective insertion requires the integration of anatomical knowledge, ultrasound interpretation, aseptic technique, technical dexterity, and real-time clinical judgement. Simulation-based training improves technical performance, reduces procedural complications, and enhances patient safety in this context,^4^^,^^5^ and is therefore widely embedded in CVAD education. However, simulation itself is not stress-neutral. Physiological stress responses, including elevated cortisol and heart rate, are well demonstrated in trainees undergoing simulation-based assessment,^6^^,^^7^ and may be pronounced in less experienced operators. Simulation therefore provides both a training platform and a useful controlled model for examining the effects of stress on procedural performance under controlled conditions.

Mindfulness-based interventions have emerged as potential strategies to reduce stress and improve attentional regulation in healthcare settings.^8^^,^^9^ Mindfulness may also exert short-term effects through attentional control and reduced physiological reactivity to stress,^10^^,^^11^ including acute reductions in heart rate, blood pressure, and cortisol.^12^ Despite this rationale, most mindfulness-based interventions studied in healthcare involve structured programmes delivered over several weeks, and a recent systematic review and meta-analysis of 99 randomised controlled trials (RCTs, N = 16,054) found that mindfulness-based interventions improved task performance compared with passive controls, but not when compared with active attentional controls,^13^ highlighting uncertainty surrounding the effectiveness of brief, immediately deployable interventions at the point of care. We therefore conducted a pilot feasibility RCT to evaluate the delivery of a brief preprocedural mindfulness-based intervention prior to simulated ultrasound-guided CVAD insertion, and to explore its effects on procedural performance, cognitive workload, psychological state, and physiological stress responses.

## 2 Methods

### 2.1 Study design and setting

This was a single-centre, parallel-group, pilot randomised controlled feasibility study conducted at the Epworth Richmond Clinical Simulation Centre, Melbourne, Australia. Governance with ethics oversight approval was obtained from the Epworth Institute Review Panel (reference: EH2025-1363). This study is reported in accordance with the Consolidated Standards of Reporting Trials (CONSORT) 2010 statement and its extension to randomised pilot and feasibility trials.^14^

### 2.2 Participants

Adult ICU registrars with prior experience in CVAD insertion were eligible. Participants were required to be aged 18 years or older and provide written informed consent. Exclusion criteria included regular mindfulness or meditation practice (≥1 h per week over the preceding 6 months), previous participation in mindfulness-performance research, or inability to safely perform the simulated procedure.

### 2.3 Consent and randomisation

Written informed consent was obtained by a researcher who was not in a direct supervisory relationship with participants. After baseline assessments, participants were randomly allocated in a 1:1 ratio to either the mindfulness intervention or active control group using computer-generated permuted block randomisation with small, fixed block sizes appropriate to the sample size. The allocation sequence was generated by a member of the research team not involved in participant recruitment, consent, or outcome assessment. Group assignments were prepared in advance and concealed from investigators until the point of intervention delivery.

### 2.4 Intervention and control

The intervention consisted of a 3-min recorded mindfulness meditation delivered immediately before the simulation task. The recording was developed in collaboration with yoga instructor Eliza Hilmer (Feel Good Flow) and focused on breath awareness and attentional regulation, structured as: 0–45 s of physical settling and present-moment awareness; 45 s to 2 min of paced diaphragmatic breathing; and 2–3 min of intentional attentional preparation for the upcoming task. This purpose-designed preprocedural mindfulness intervention incorporates core components (breath awareness, body scan, nonjudgemental attention) with established mechanistic support. Existing validated protocols were not used, as they typically require 10–45 min and are not feasible in the immediate preprocedural setting. In addition, there are no validated brief mindfulness-based intervention (≤5-min) exists for this context (Supplementary File 1).

The active control group completed a time-matched (3-min) review of a standardised single-page observation chart depicting physiological parameters for a generic postoperative patient. The chart contained no mindfulness content and was selected to provide an active attentional comparator that controlled for time and the provision of a structured pre-procedural activity without inducing a relaxation or mindfulness response. Both conditions were delivered under identical environmental conditions (Supplementary File 2).

### 2.5 Simulation procedure

Participants performed ultrasound-guided right internal jugular CVAD insertion in a standardised simulation scenario involving a postoperative patient requiring vasopressor support. The scenario, equipment configuration, and task sequence were standardised across participants using a preprepared procedure trolley and scripted briefing. All procedures were video-recorded with fixed camera positioning for subsequent blinded performance assessment (Supplementary File 5).

### 2.6 Feasibility outcomes

The primary outcome was feasibility, assessed against prespecified thresholds a priori. Feasibility was considered achieved if: (i) ≥80% of eligible participants were recruited; (ii) ≥90% adhered to the intervention (defined as completion of the assigned preprocedural activity and the simulation task); (iii) ≥95% outcome data completeness was achieved; and (iv) assessor blinding was maintained throughout. These thresholds were based on established standard pilot study methodology.^14^^,^^15^

### 2.7 Exploratory outcome measures

Exploratory outcomes included procedural performance, physiological measures, and psychological metrics. Procedural performance was assessed using a task-specific 15-item procedural checklist (scored 0–2 per item, maximum score 30), a 6-domain global rating scale score (GRS; scored 1–5 per domain, maximum score 30) and a bespoke 12-item nontechnical performance observational tool assessing patient discomfort recognition, monitoring, and situational awareness behaviours (scored 0–2 per item, maximum score 24; developed for this study, not independently validated) (Supplementary File 3). The procedural checklist and GRS were developed for this study through iterative expert review by experienced ICU clinicians and simulation educators and were piloted prior to the study. The total performance score combined checklist and GRS scores (maximum 60). Predefined critical procedural errors were recorded prospectively.

Physiological measures included heart rate and systolic blood pressure measured at baseline, immediately postintervention and immediately postsimulation. Cognitive workload was assessed using a modified four-domain NASA Task Load Index (NASA-TLX, scored 0–20 per domain with a total range of 0–80).^16^ State anxiety was assessed using a four-item abbreviated measure derived from the Spielberger State-Trait Anxiety Inventory State subscale (STAI-State; scored 4–16), with higher scores indicating greater anxiety.^17^ Self-reported procedural confidence was assessed using a four-item study-specific visual analogue scale (scored 4–20) with higher scores indicating greater confidence (Supplementary File 4).

### 2.8 Performance assessment

Video-recorded procedures were assessed by two independent assessors blinded to group allocation, following calibration with training videos. Assessor-level scores were not retained in the study dataset, precluding formal inter-rater reliability quantification; this has been acknowledged as a limitation. Reported performance outcomes were based on the available assessor ratings.

### 2.9 Statistical analysis

Given the pilot nature of the study and the intentionally small sample size, all analyses were descriptive. Continuous variables are presented as mean and standard deviations (SDs) or median and interquartile ranges (IQRs). Categorical variables are presented as counts and percentages. For the psychological domain panel, group means and 95% confidence intervals (95% CIs) were computed at each timepoint. Outcomes were summarised by treatment group to estimate feasibility, variability and signal of effect. No formal hypothesis testing was undertaken, consistent with pilot feasibility study reporting guidelines.^14^^,^^15^

### 2.10 Ethics approval and consent to participate

The study was approved by the Epworth Human Research Ethics Committee (reference: EH2025-1363) under the Non-HREC Pathway for low-risk research. Written informed consent was obtained from all participants prior to any study procedures.

## 3 Results

### 3.1 Participant flow

**Fig. 1:**
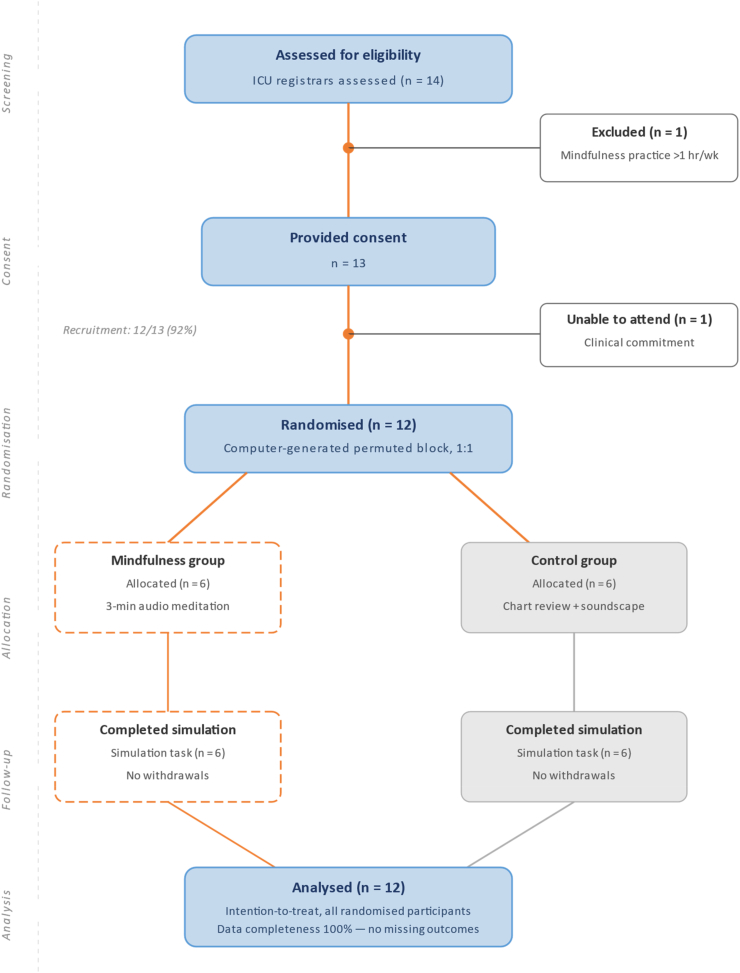
CONSORT participant flow diagram. CONSORT, Consolidated Standards of Reporting Trials.

Thirteen participants provided informed consent; of these, one was subsequently unable to attend due to a clinical commitment. Twelve participants were enrolled, consented, and randomised (six per group). All 12 completed the session; no withdrawals, protocol deviations, or adverse events occurred. Fig. 1 shows the CONSORT participant flow.

### 3.2 Participant characteristics

Baseline characteristics were broadly comparable (Table 1). Both groups had comparable age distributions with equal proportions of junior and senior registrars. Both groups had comparable procedural experience. The baseline mean heart rate in the mindfulness group was relatively higher (82.7 [±10.3] bpm) compared with the control group (77.3 [±8.6] bpm). The systolic blood pressure was comparable between the 2 groups.

**Table 1:** Baseline characteristics of participants by group.

Characteristic	Mindfulness (n = 6)	Control (n = 6)
**Age range, n (%)**		
25–34 years	2 (33%)	2 (33%)
35–44 years	3 (50%)	3 (50%)
45–54 years	1 (17%)	1 (17%)
**Sex, n (%)**		
Male	4 (67%)	4 (67%)
Female	2 (33%)	2 (33%)
**Professional role, n (%)**		
Junior ICU registrar (PGY 3–4)	3 (50%)	3 (50%)
Senior ICU registrar (PGY ≥5)	3 (50%)	3 (50%)
**Years postgraduate experience, n (%)**		
3–5 years	1 (17%)	2 (33%)
6–10 years	1 (17%)	1 (17%)
>10 years	4 (67%)	3 (50%)
**Prior CVAD insertions, n (%)**		
0–10	1 (17%)	1 (17%)
11–30	3 (50%)	3 (50%)
>30	2 (33%)	2 (33%)
**Prior simulation-based CVAD assessment, n (%)**	2 (33%)	2 (33%)
**Baseline heart rate, mean (SD), bpm**	82.7 (10.3)	77.3 (8.6)
**Baseline systolic BP, mean (SD), mmHg**	133.8 (14.2)	135.0 (11.8)
**Baseline state anxiety**^a^**, mean (SD)**	10.5 (2.8)	8.7 (2.1)
**Anticipated NASA-TLX total**^b^**, mean (SD)**	37.2 (9.6)	43.8 (11.2)

CVAD = central venous access device; BP = blood pressure; bpm = beats per minute; SD = standard deviation; NASA-TLX = NASA Task Load Index.

a 4-item abbreviated STAI-State, scored 4–16; higher scores indicate greater state anxiety.

b 4-domain modified NASA-TLX, scored 0–80; higher scores indicate greater perceived workload.

### 3.3 Primary outcome: feasibility

All prespecified feasibility thresholds were met. Recruitment was 92% (12/13, exceeding the ≥80% threshold). Intervention adherence was 100% (6/6 per group, exceeding ≥90%). Outcome data completeness was 100% across all instruments and timepoints (exceeding ≥95%). Assessor blinding was maintained throughout with no unblinding events.

### 3.4 Exploratory outcomes

(i) Procedural performance

**Fig. 2:**
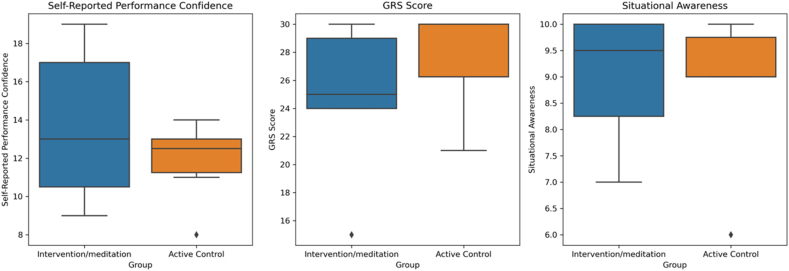
Box plot distributions of procedural performance measures (total performance score, checklist score, and GRS score) and self-reported confidence, by group. Box plots show the median (horizontal line), interquartile range (box), and range (whiskers) for the mindfulness intervention group (blue) and active control group (orange). Total performance score (maximum 60) was lower in the mindfulness group (mean 41.3 vs 52.0). Procedural checklist score (maximum 30) was also lower in the mindfulness group (mean 16.5 vs 24.3), with a wider spread. GRS score (maximum 30) showed a modest difference (mean 24.8 vs 27.7). Self-reported postsimulation confidence (scored 4–20) was slightly higher in the mindfulness group (mean 13.7 vs 11.8). All differences should be interpreted as exploratory given the small sample size (n = 6 per group). GRS, global rating scale. (For interpretation of the references to colour in this figure legend, the reader is referred to the web version of this article.)

Procedural performance was numerically lower in the mindfulness group across all measures (Fig. 2, Table 2). Mean checklist score was 16.5 [±9.5] vs 24.3 [±8.8] in the control group; mean GRS scores were 24.8 [±5.8] vs 27.7 [±3.8], respectively, resulting in a lower total mean performance score in the mindfulness group (41.3 [±15.1]) compared with the control group (52.0 [±12.4]). Although the absolute situational awareness score was low overall, it was comparable between the two groups (9.0 vs 8.8). Self-reported procedural confidence was modestly higher in the intervention group when compared with the control group (mean 13.7 vs 11.8). Of note, critical procedural errors occurred substantially more frequently in the mindfulness group (66.7% [4/6] vs 16.7% [1/6]). Errors included arterial puncture (n = 2, mindfulness group), loss of guidewire control (n = 1 in each group), and failure to confirm venous placement prior to use (n = 1, mindfulness group). Arterial puncture in particular represents a clinically significant error with direct patient safety implications. The concentration of errors in the mindfulness group is notable; however, with only six participants per group, the distribution is susceptible to chance imbalance. Both arterial punctures occurred in participants who also had higher baseline heart rates and anxiety scores, raising the possibility that pre-existing physiological differences between groups may have contributed.

(ii) Physiological measures

**Table 2:** Exploratory outcome measures by group.

Outcome	Mindfulness (n = 6)	Control (n = 6)
**Procedural performance**		
Checklist score (max 30), mean (SD)	16.5 (9.5)	24.3 (8.8)
GRS score (max 30), mean (SD)	24.8 (5.8)	27.7 (3.8)
Total performance score (max 60), mean (SD)	41.3 (15.1)	52.0 (12.4)
Situational awareness score (max 24), mean (SD)	9.0 (1.3)	8.8 (1.5)
Critical procedural errors, n participants affected	4/6 (67%)	1/6 (17%)
**Physiological measures**		
Baseline heart rate, mean (SD), bpm	82.7 (10.3)	77.3 (8.6)
Post-intervention heart rate, mean (SD), bpm	70.3 (9.1)	72.0 (7.8)
Post-simulation heart rate, mean (SD), bpm	83.7 (11.4)	75.7 (9.2)
Baseline systolic BP, mean (SD), mmHg	133.8 (14.2)	135.0 (11.8)
Post-intervention systolic BP, mean (SD), mmHg	120.3 (12.6)	131.3 (10.4)
**Psychological measures and workload**		
Baseline state anxiety^a^, mean (SD)	10.5 (2.8)	8.7 (2.1)
Post-intervention state anxiety^a^, mean (SD)	8.7 (1.3)	8.7 (2.3)
Pre-simulation NASA-TLX total^b^, mean (SD)	37.2 (9.6)	43.8 (11.2)
Post-simulation NASA-TLX total^b^, mean (SD)	37.2 (10.1)	43.3 (10.8)
Mental demand^c^, baseline/postintervention	9.2/10.2	11.8/12.2
Time pressure^c^, baseline/postintervention	9.2/8.5	11.8/8.3
Effort required^c^, baseline/postintervention	10.8/9.0	11.5/13.3
Frustration level^c^, baseline/postintervention	8.0/9.5	8.7/9.5
Post-simulation confidence^d^, mean (SD)	13.7 (3.2)	11.8 (2.9)

GRS = global rating scale; BP = blood pressure; bpm = beats per minute; SD = standard deviation; NASA-TLX = NASA Task Load Index.

a 4-item abbreviated STAI-State, scored 4–16.

b 4-domain modified NASA-TLX, scored 0–80.

c Individual NASA-TLX domain, scored 0–20; means only reported given descriptive sample size.

d 4-item study-specific confidence scale, scored 4–20.

**Fig. 3:**
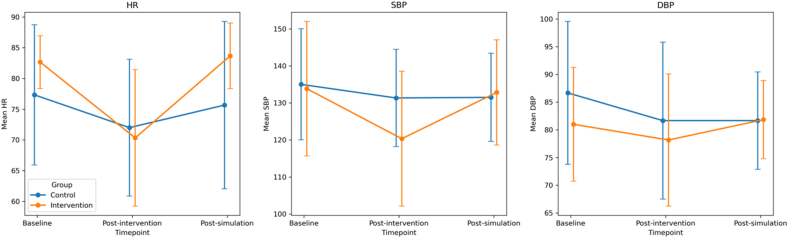
Physiological measures across three time points by group. The error bars represent 95% CI. Mean heart rate (bpm) and systolic blood pressure (mmHg) at baseline, postintervention, and postsimulation for the mindfulness intervention (n = 6) and active control (n = 6) groups. Heart rate decreased more following the mindfulness intervention than the control (−12.4 vs −5.3 bpm), but returned to above-baseline levels postsimulation in the mindfulness group (83.7 vs 75.7 bpm). Systolic blood pressure showed a similar pattern, with a greater post-intervention reduction in the mindfulness group (−13.5 vs −3.7 mmHg) but comparable postsimulation values between groups. All values are means; no formal inferential statistics were performed, consistent with the pilot and descriptive nature of the study. CI, confidence interval.

Heart rate variation was transient. Although it decreased more postintervention in the mindfulness group (−12.4 vs −5.3 bpm), the postsimulation heart rate was higher (83.7 vs 75.7 bpm). Systolic and diastolic blood pressure showed a similar pattern, with a greater postintervention reduction in the mindfulness group, but comparable postsimulation values (Fig. 3).

(iii) Psychological measures and workload

**Fig. 4:**
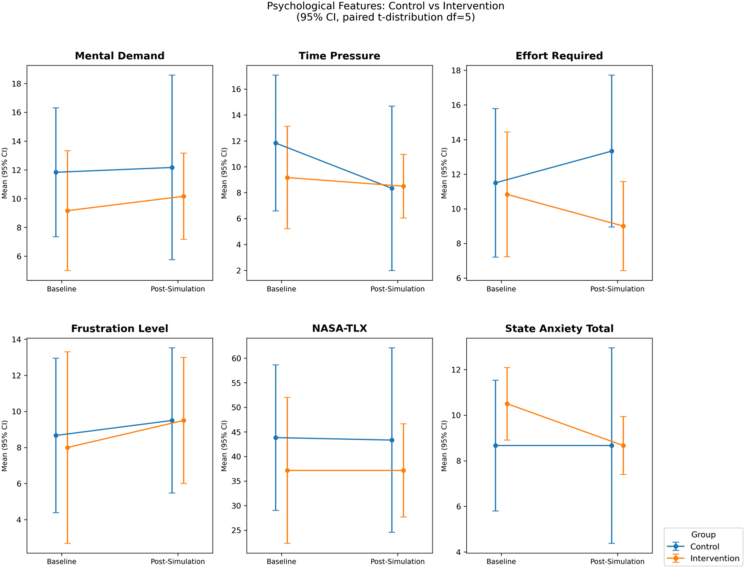
Faceted panel plot of six psychological outcome measures across baseline and postsimulation time points, by group. The error bars represent 95% CIs (paired t-distribution, df = 5). Mean scores and 95% confidence intervals for six psychological outcome measures (Mental Demand, Time Pressure, Effort Required, Frustration Level, NASA-TLX total, and State Anxiety Total) at baseline and postsimulation, shown separately for the control (n = 6) and mindfulness intervention (n = 6) groups. Confidence intervals were calculated using the t-distribution (df = 5, t-critical = 2.571) applied to individual participant data at each time point independently. The most directionally consistent finding was a reduction in State Anxiety in the mindfulness group (10.5–8.7), while it remained essentially unchanged in controls, resulting in convergence at postintervention. Effort required showed a divergent pattern (mindfulness: 10.8 to 9.0; control: 11.5 to 13.3), directionally consistent with differing cognitive engagement profiles of the two preparatory conditions. All other domains showed parallel or near-parallel trajectories. Confidence intervals were wide and overlapped considerably throughout, consistent with the exploratory nature of these analyses. CIs, confidence intervals.

Psychological outcomes are summarised in Fig. 4. All findings are exploratory, with wide and considerably overlapping confidence intervals. State anxiety showed the most consistent directional change, decreasing in the mindfulness group (10.5–8.7) while remaining unchanged in controls. Effort required showed divergent patterns from baseline to postsimulation (mindfulness group: 10.8 to 9.0; control group: 11.5 to 13.3), consistent with differing cognitive demands. Other domains (mental demand, time pressure, frustration level, and total workload) showed similar trajectories between groups.

## 4 Discussion

This pilot randomised feasibility study demonstrated that a brief preprocedural mindfulness-based intervention can be successfully integrated into a single-session simulation workflow, meeting all prespecified feasibility thresholds. A brief preprocedural mindfulness-based intervention produced measurable, albeit transient, physiological and psychological effects; however, procedural performance scores were numerically lower, with more critical errors in the mindfulness group. Our findings highlight a potentially important interaction between arousal reduction and procedural readiness that warrants further investigation in an adequately powered trial.

Feasibility outcomes were robust. Recruitment was high (92%), with complete adherence, full data capture, and no adverse events. Assessor blinding was also preserved through standardised video-based assessment, supporting internal validity. This 3-min mindfulness-based intervention was seamlessly embedded into a time-pressured training environment, supporting its acceptability and practicality. These attributes, such as brevity, low resource requirement, and ease of delivery, suggest that this mindfulness-based intervention is potentially scalable and adaptable beyond the index procedure across critical care, perioperative medicine, and emergency settings. However, scalability across diverse institutions and clinical environments requires evaluation in a multicentre design.

Simulation-based training has been shown to improve clinical outcomes for CVAD insertion, including reduced complication rates.^18^ However, the extent to which performance in a simulated environment translates to real-world procedural performance remains influenced by factors such as task fidelity and observer effects. Importantly, the transferability of psychological interventions, such as mindfulness, from simulation to clinical practice has not been established and warrants further study.

Exploratory findings suggested modest, transient reductions in physiological arousal following the intervention, consistent with prior evidence demonstrating attenuation of acute sympathoadrenal responses with brief meditation practices in procedural and surgical contexts.^12^ These changes were accompanied by small directional improvements in subjective measures, such as reduced anxiety and perceived effort, and slightly higher confidence. However, other psychological domains, including mental demand, time pressure, frustration level, and overall NASA-TLX scores, showed parallel or near-parallel trajectories between groups, with no consistent differential effect. Furthermore, these physiological and psychological effects did not translate into improved procedural performance.

Importantly, we observed a dissociation between physiological/psychological effects and performance, with lower procedural scores and more errors in the mindfulness group (4/6 vs 1/6), albeit in a small sample. This pattern was consistent with a recent systematic review and meta-analysis that found mindfulness-based interventions did not outperform active controls on task performance.^13^ One explanation is the Yerkes–Dodson inverted-U relationship between arousal and performance.^3^ The observed reduction in heart rate may have shifted some participants below the optimal arousal threshold for complex psychomotor performance. In this context, a state of relative hypo-arousal may be subjectively calming, consistent with higher confidence, yet procedurally suboptimal.

The transient nature of the physiological effect, with higher postsimulation heart rates in the mindfulness group, suggests a mismatch between the preparatory state and task demands. Selection into critical care may also confer higher baseline arousal, resulting in a right-shifted optimal performance zone. Within this framework, arousal-reduction interventions may have heterogeneous effects depending on baseline arousal state. We therefore propose a hypothesis-generating arousal-performance model (Supplementary File 6), suggesting that mindfulness may overshoot the optimal zone in some individuals, contributing to a confidence-competence dissociation. These findings are exploratory and should be interpreted cautiously.

From a clinical perspective, the possibility that a brief mindfulness-based intervention may transiently reduce vigilance or attentional readiness warrants particular attention, given its direct implications for patient safety, and should be a key consideration in the design of future trials. Although causality cannot be inferred, the higher rate of critical errors, including arterial punctures, in the mindfulness group raises potential patient safety considerations. This possibility is consistent with established literature demonstrating that self-perceived confidence and observed competence frequently correlate poorly in health professions education.^19^ These findings underscore the importance of incorporating objective, assessor-rated outcomes in future trials and highlight the need to carefully evaluate the timing, duration, and target population for such interventions.

Alternative explanations should also be considered. The lack of stratification by baseline procedural skill or formal CVAD competency certification status may have introduced chance imbalance between groups. The active control may have functioned more as a passive rest condition, potentially reducing its validity as a comparator. Differences in intervention duration, task complexity, and participant characteristics may also explain discrepancies with prior studies demonstrating performance benefits with longer mindfulness interventions.^20^ Collectively, these factors reinforce that the current findings are hypothesis-generating rather than definitive.

### 4.1 Strengths and limitations

This study has several strengths. It used a prospective randomised pilot design within a clinically relevant simulation task, and incorporated objective structured assessment of procedural performance alongside physiological and psychological measures. The study was successfully implemented within a pragmatic acute care education environment, supporting practical feasibility.

The study also has important limitations. First, this single-centre study conducted in a cohort of ICU registrars at a private tertiary hospital may limit generalisability across settings and experience levels and precludes assessment of scalability across different ICU training environments. Second, the sample size limits estimation of effect sizes and variability and therefore constrains planning for future trials. As a pilot study, it was designed to assess feasibility metrics rather than provide precise estimates of treatment effects. Third, the simulation environment, while standardised, cannot fully replicate the complexity and pressures of real-world clinical care. Fourth, observer-induced bias (Hawthorne effect) may have differentially influenced performance across groups; for example, participants in a calmer physiological state may exhibit different behavioural responses to observation. Fifth, no baseline procedural performance data were collected, therefore, between-group baseline differences in procedural skill could not be excluded despite randomisation. Sixth, the active control appeared suboptimal based on participant feedback, suggesting that it may have functioned more as a passive rest condition than a true active attentional comparator, which may have reduced internal validity. Critically, inter-rater reliability for procedural performance could not be formally assessed, as assessor-level data were unavailable; all reported performance scores therefore derive from a single assessor's ratings of unverified reliability, representing an important methodological limitation that should be prioritised in future trials. Seventh, given that this mindfulness-based intervention has not been independently validated in other settings; its reproducibility and fidelity in different contexts require evaluation. Eighth, formal CVAD competency certification status was not recorded. Finally, the study was not prospectively registered on a clinical trials registry; prospective registration will be obtained prior to any subsequent trial.

### 4.2 Implications and future directions

These pilot data establish feasibility and provide a clear framework for the design of a multicentre, adequately powered randomised trial to determine whether brief mindfulness-based interventions can meaningfully influence performance or are better positioned as tools for stress modulation and clinician preparedness. The psychological findings from our pilot suggest that state anxiety may represent a measurable mechanistic signal and should be included as a prespecified secondary outcome with appropriate instrument selection and timing.

The intervention itself was brief, low-cost, and simple to deliver. These attributes support potential scalability within acute care education environments, where longer structured programmes are often impractical.[14], [15], [16] From a translational perspective, although this study was conducted in a simulation environment, these characteristics support potential applicability in clinical practice. If shown to be effective, particularly in appropriately selected individuals, such strategies may offer a simple and scalable approach to modulating clinician stress and preparedness in high-acuity patient care contexts.

## 5 Conclusion

This pilot RCT met all prespecified feasibility thresholds, demonstrating that a brief preprocedural mindfulness-based intervention can be delivered within a simulation-based ICU training workflow. This intervention produced transient reductions in physiological and psychological measures, but procedural performance scores were numerically lower in the mindfulness group across all domains, and critical errors were more frequent, raising the possibility that arousal reduction may have shifted participants below the optimal vigilance zone for complex procedural tasks. These findings support progression to a larger, multicentre trial with prespecified safety monitoring of performance outcomes.

## CRediT authorship contribution statement

DOD conceived and designed the study, collected data, performed analyses, and drafted the manuscript. AS, TK and LP contributed to study design, methodology, statistical analysis plan, and critically revised the manuscript for important intellectual content. DM and LP designed and performed the statistical analysis. All authors approved the final version for submission and agree to be accountable for all aspects of the work.

## Trial registration

This pilot study was not prospectively registered on a clinical trials registry. This is acknowledged as a limitation.

## Data availability

De-identified study data may be made available from the corresponding author on reasonable request, subject to ethics approval.

## Funding

No external funding was received for this study. The mindfulness audio recording was developed pro bono by Eliza Hilmer (Feel Good Flow).

## Conflict of interest

The authors declare the following financial interests/personal relationships which may be considered as potential competing interests: Ashwin Subramaniam reports a relationship with Serves as an associate editor for Critical Care and Resuscitation and is the Intensive Care Medicine subspecialty editor for Internal Medicine Journal that includes: employment. If there are other authors, they declare that they have no known competing financial interests or personal relationships that could have appeared to influence the work reported in this paper.
