# Supplementary material for: Brief mindfulness intervention prior to simulated central venous access device insertion: A pilot randomised feasibility study
Source: Crit Care Resusc. 2026 Jul 9;28(3):100198. doi: 10.1016/j.ccrj.2026.100198 (PMC13376986; doi:10.1016/j.ccrj.2026.100198)
Supplement: Multimedia component 1 [file mmc1.docx]

***Supplementary Materials***

**Supplementary File 1.** Mindfulness intervention audio recording transcript (3-minute breath awareness and attentional regulation script developed in collaboration with Eliza Hilmer, Feel Good Flow).

“Let’s begin by letting your attention settle into the present moment.
Gently close your eyes if that feels comfortable, and notice the sensation of your feet grounded on the floor and your body supported by the chair.

Bring awareness to your breathing. Take a slow inhale through your nose... and then a longer, slower exhale out through your mouth. Inhale... and exhale for just a little longer.

Feel your body steadying as you lengthen the exhalation. This longer out-breath signals safety to the nervous system and helps regulate the stress response.

Now bring to mind the central venous catheter procedure you’ll be performing shortly. Imagine yourself preparing equipment with clarity and composure.

Picture identifying the anatomy, making deliberate movements, and attending to each step smoothly and efficiently.

Notice yourself responding to challenges with focus and with an even tempo. If a moment of stress arises, see yourself returning to that longer exhale, steadying your attention and your hands.

Imagine guiding the needle, threading the wire, and placing the catheter with control and confidence. See the whole sequence unfolding with precision and calm focus.

As you hear my voice, begin to connect the technique you’ve practiced, the longer exhalation with the procedure ahead.

During the simulation, if you feel tension or pressure building, simply return to that long, slow out-breath to help steady your system and maintain situational awareness.

Take a final slow inhale... and an even longer exhale.
And when you’re ready, open your eyes and bring that sense of calm concentration forward with you.”

**Supplementary File 2.** Active control stimulus: standardised single-page observation chart used in the control condition.

**Supplementary File 3.** CVAD procedural performance instruments: 15-item procedural checklist (scored 0-2 per item; maximum 30), 6-domain Global Rating Scale (scored 1-5 per domain; maximum 30), and 12-item situational awareness tool (maximum 24), with assessor instructions and anchor descriptors.

Procedural Checklist

Each item scored as:
**0 = Not performed / Incorrect**
**1 = Performed inadequately or out of sequence**
**2 = Performed correctly and appropriately**

**🡪 Termination of task if inserts into artery or inappropriate/unsafe technique**

| Step | Procedural Element | Score (0-2) |
| --- | --- | --- |
| 1 | Confirms indication for CVC and checks patient identity | ☐0 ☐1 ☐2 |
| 2 | Performs hand hygiene and dons appropriate PPE | ☐0 ☐1 ☐2 |
| 3 | Prepares and maintains full aseptic field | ☐0 ☐1 ☐2 |
| 4 | Uses ultrasound to correctly identify IJ vein and surrounding anatomy | ☐0 ☐1 ☐2 |
| 5 | Confirms vein patency and avoids arterial puncture on ultrasound | ☐0 ☐1 ☐2 |
| 6 | States they will administer local anaesthetic appropriately | ☐0 ☐1 ☐2 |
| 7 | Needle insertion with correct technique and venous aspiration | ☐0 ☐1 ☐2 |
| 8 | Guidewire advanced smoothly without resistance | ☐0 ☐1 ☐2 |
| 9 | Guidewire position confirmed (ultrasound/aspiration) | ☐0 ☐1 ☐2 |
| 10 | Dilator used appropriately with haemostasis maintained | ☐0 ☐1 ☐2 |
| 11 | Catheter advanced to appropriate depth for patient height | ☐0 ☐1 ☐2 |
| 12 | Guidewire removed safely and completely | ☐0 ☐1 ☐2 |
| 13 | All lumens aspirated and flushed correctly | ☐0 ☐1 ☐2 |
| 14 | Venous blood gas checked and confirms venous placement | ☐0 ☐1 ☐2 |
| 15 | States will secure appropriately (sutures/dressing) and order CXR | ☐0 ☐1 ☐2 |

**Checklist Subtotal (max 30):** ______ /30

## Global Rating Scale (GRS)

Each domain scored from **1 (poor) to 5 (excellent)**

| Domain | 1 | 2 | 3 | 4 | 5 |
| --- | --- | --- | --- | --- | --- |
| Respect for aseptic technique | ☐ | ☐ | ☐ | ☐ | ☐ |
| Instrument handling and flow of procedure | ☐ | ☐ | ☐ | ☐ | ☐ |
| Ultrasound use and interpretation | ☐ | ☐ | ☐ | ☐ | ☐ |
| Efficiency and time management | ☐ | ☐ | ☐ | ☐ | ☐ |
| Situational awareness and error avoidance | ☐ | ☐ | ☐ | ☐ | ☐ |
| Overall procedural competence | ☐ | ☐ | ☐ | ☐ | ☐ |

**GRS Subtotal (max 30):** ______ /30

Patient Discomfort, Monitoring & Situational Awareness Scoring Section

Observed Behaviours During Discomfort Events

Behaviour Score (0-2)

Recognises patient distress/discomfort ☐ 0 ☐ 1 ☐ 2

Provides reassurance & clear communication ☐ 0 ☐ 1 ☐ 2

Checks patient observations (HR/BP/SpO₂) when indicated ☐ 0 ☐ 1 ☐ 2

Temporises/pauses procedure when appropriate ☐ 0 ☐ 1 ☐ 2

Explains procedural sensations (e.g., “you may feel pressure now”) ☐ 0 ☐ 1 ☐ 2

**Scenario triggers included:**
- draping dyspnoea (“I can’t breathe”)
- pain during local anaesthetic infiltration
- anxiety/discomfort during jugular dilation

**Scoring key:**
**2 = appropriate & consistent**, **1 = partial/inconsistent**, **0 = absent/inappropriate**

**Supplementary File 4.** Psychological outcome instruments: 4-item abbreviated STAI-State (scored 4-16), 4-domain modified NASA Task Load Index (scored 0-80), and 4-item procedural confidence visual analogue scale (scored 4-20), with administration instructions.

### State Anxiety (STAI-S Abbreviated Version)

Instructions: Please indicate how you feel right now, at this moment.

Rate each item from 1 (Not at all) to 4 (Very much so).

| Item | 1 | 2 | 3 | 4 |
| --- | --- | --- | --- | --- |
| I feel calm | ☐ | ☐ | ☐ | ☐ |
| I feel tense | ☐ | ☐ | ☐ | ☐ |
| I feel nervous | ☐ | ☐ | ☐ | ☐ |
| I feel relaxed | ☐ | ☐ | ☐ | ☐ |

### Section 3: Anticipated Cognitive Load (NASA-TLX - Modified)

Please rate your anticipated workload for the upcoming task.

Scale: 0 (Very Low) - 20 (Very High)

- Mental Demand: ___ /20
- Time Pressure: ___ /20
- Effort Required: ___ /20
- Frustration Level: ___ /20

**Supplementary File 5.** Simulation scenario briefing script and equipment configuration checklist for standardised ultrasound-guided right internal jugular CVAD insertion.

### *Simulation Setting and Clinical Context*

The simulation involves a 76-year-old male admitted to a high-dependency or intensive care setting following revision total knee arthroplasty. His past medical history includes ischaemic heart disease with prior myocardial infarction requiring percutaneous coronary intervention with two coronary stents (2018), atrial fibrillation (with perioperative interruption of anticoagulation), type 2 diabetes mellitus, hypertension, and prior right total knee replacement. Ex AFL player.

In the immediate postoperative period, the patient demonstrates haemodynamic instability, characterised by hypotension requiring escalating doses of peripheral metaraminol. Surgical drain output is increased; however, there is no significant reduction in haemoglobin, and there are no features of overt haemorrhagic shock. The clinical picture is consistent with postoperative vasoplegia or distributive shock requiring central vasopressor support.

Given the requirement to commence a continuous noradrenaline infusion, insertion of a central venous catheter (CVC) is indicated for safe administration of vasoactive therapy and haemodynamic monitoring.

### *Simulation Task*

Participants are required to perform ultrasound-guided internal jugular vein central venous catheter insertion in a simulated environment. The procedure trolley is pre-prepared by an assistant nurse to standardise equipment availability and reduce variability.

### *Procedural Requirements*

Participants must demonstrate the following procedural steps, consistent with best clinical practice:

1. Identification of relevant anatomy using real-time ultrasound guidance
2. Establishment of full aseptic technique and sterile field
3. Administration of local anaesthetic
4. Needle insertion with aspiration of venous blood from the internal jugular vein
5. Passage of guidewire with confirmation of intravascular positioning using ultrasound
6. Confirmation of venous placement via venous blood gas analysis
7. Tract dilation with attention to minimising blood loss
8. Advancement of the central venous catheter over the guidewire using the Seldinger technique, to an appropriate depth based on patient height
9. Removal of the guidewire
10. Securement of the catheter
11. Review of post-procedure chest radiograph to confirm catheter position and exclude complications prior to use

This simulation scenario is designed to reflect a realistic, clinically relevant indication for CVC insertion under time pressure, while allowing standardisation of task complexity, environment, and assessment conditions across participants.

**Supplementary File 6:** Hypothesised arousal-performance quadrant model based on the Yerkes-Dodson law.

*
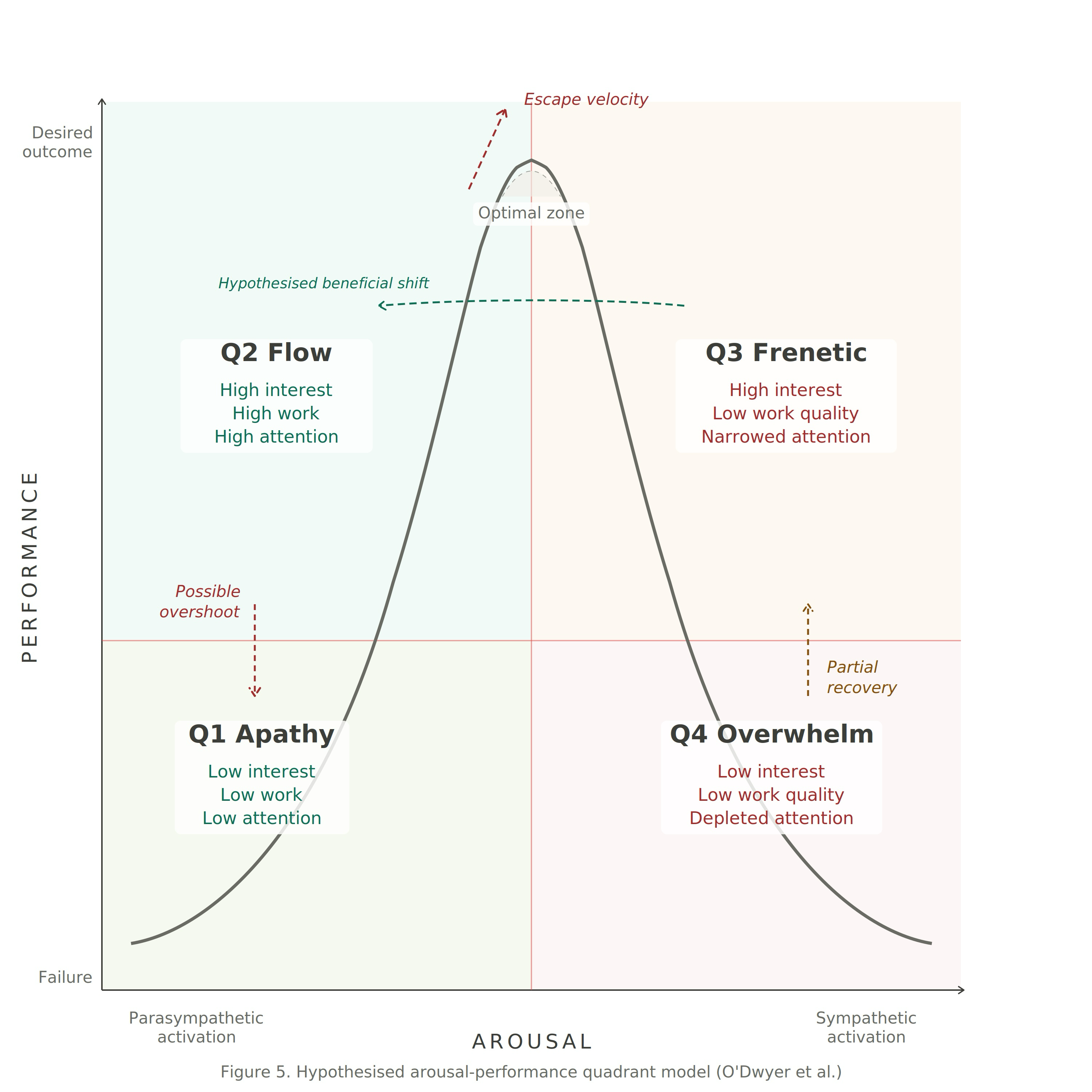
*

The model proposes four quadrants: Q1 (apathy: low arousal, low performance, characterised by reduced interest, work, and attention), Q2 (flow: moderate arousal, high performance, characterised by high interest, sustained work, and focused attention), Q3 (frenetic: high arousal, variable performance, characterised by sustained interest but narrowed attention and reduced work quality), and Q4 (overwhelm: excessive arousal, low performance, characterised by depleted interest, work quality, and attention). Dashed arrows indicate hypothesised intervention effects: desired shift from Q3 to Q2 (green), possible overshoot to Q1 (red), and partial recovery from Q4 to Q3 (amber). The “escape velocity” (top red arrow) represents sustained optimal performance. This model is hypothesis-generating and requires validation in a larger cohort.
